# A New Atisane-Type Diterpene from the Bark of the Mangrove Plant *Excoecaria Agallocha*

**DOI:** 10.3390/molecules14010414

**Published:** 2009-01-16

**Authors:** Zhan Chang Wang, Yi Ming Lin, Dan Qin Feng, Cai Huan Ke, Peng Lin, Chong Ling Yan, Jun De Chen

**Affiliations:** 1 Key Laboratory of Ministry of Education for Coastal and Wetland Ecosystems, School of Life Sciences, Xiamen University, Xiamen 361005, China; 2 College of Oceanography and Environmental Science, Xiamen University, Xiamen 361005, China; 3 The Third Institute of Oceanography State Oceanic Administration, Xiamen 361005, China

**Keywords:** Diterpenes, Biofilm, *Excoecaria agallocha*, Anti-microfouling.

## Abstract

A new atisane-type diterpene, *ent*-16α-hydroxy-atisane-3,4-lactone (**4**) and three known diterpenes, *ent*-16α-hydroxy-atisane-3-one (**1**), *ent*-atisane-3β,16α-diol (**2**), *ent*-3,4-seco-16α-hydroxyatis- 4(19)-en -3-oic acid (**3**) were isolated from the bark of the mangrove plant *Excoecaria agallocha*. Their structures and relative stereochemistry were elucidated by means of extensive NMR and MS analysis. Compound **3** exhibited significant anti-microfouling activity against the adherence of *Pseudomonas pseudoalcaligenes*, with an EC_50_ value of 0.54 ± 0.01 ppm.

## Introduction

*Excoecaria agallocha* L (Euphorbiaceae) is an important mangrove species mainly distributed in China, India, Philippines, and Oceania [1]. This plant is used as a traditional remedy for epilepsy, conjunctivitis, dermatitis, haematuria, leprosy, and toothache [2]. The latex and leaves have been used as a dart poison and fish poison in Sarawak, New Caledonia, and Goa [3,4,5]. The phorbol ester isolated from the leaves and stems has been proved to be cytoprotective in the NCI primary anti-HIV screen [6]. Some diterpenes isolated from the wood of this plant showed anti-tumor-promoting activity [7,8]. However, there is scant information on the isolation of antifouling compounds from mangrove species [9,10].

Biofouling, the undesirable buildup of sessile marine organisms (such as barnacles, mussels, tubeworms, and seaweeds) onto man-made surfaces, causes severe problems in the maritime industry [11]. In many cases, biofouling will consist of microscopic organic impurities or a visible slimy layer containing bacteria and other microorganisms. This category of biofouling is called microfouling, or more commonly biofilm, and occurs everywhere in both natural and industrial environments where surfaces are exposed to water [12]. Once this biofilm has formed, higher ordered organisms such as barnacles, algae, mollusks, tubeworms and sponges can attach. Therefore preventing the attachment of bacteria during the early stages of biofilm formation could greatly reduce the subsequent biofouling problems [13].

**Figure 1:**
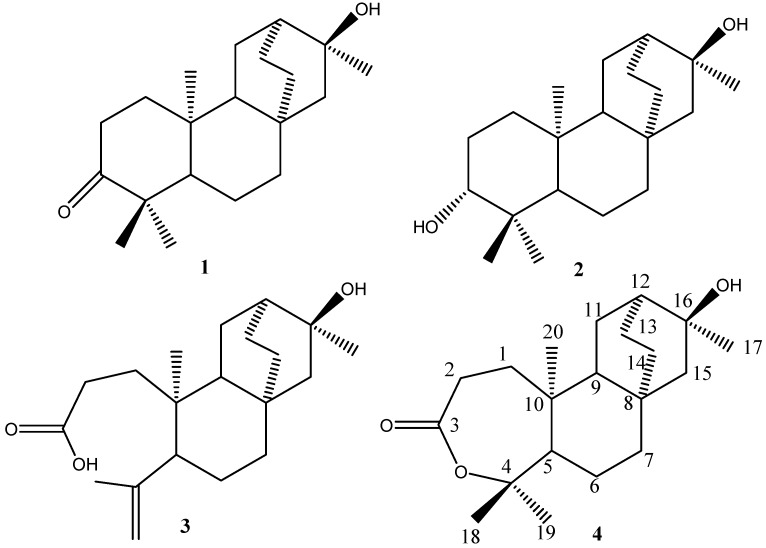
Structures of the compounds **1-4**.

Marine organisms use both physical and chemical methods to protect themselves from the harmful process of biofouling [14,15]. The key chemical antifouling mechanism of marine organisms occurs via the production of secondary metabolites (also known as natural products) which deter foulers [16]. Mangroves are unique intertidal woody communities common in tropical and subtropical coastlines [17], and as such, these plants are also affected with marine-fouling organisms [18]. Our investigation on the bark of *E. agallocha* has resulted in the isolation of a new diterpene, *ent*-16α-hydroxy-atisane-3,4-lactone (**4**), along with three known compounds, *ent*-16α-hydroxy-atisane-3-one (**1**) [19,20], *ent*-atisane-3β,16α-diol (**2**) [20], and excoecarin V3 [=*ent*-3,4-*seco*-16α-hydroxyatis-4(19)-en-3-oic acid (**3**) [21,22,23] (Figure 1). Compound **3** significantly inhibited the adherence of the marine microorganisms, *Pseudomonas pseudoalcaligenes*. The isolation, structural elucidation and anti-microfouling activity of these compounds are described in this paper.

## Results and Discussion

One new diterpene, *ent*-16α-hydroxy-atisane-3,4-lactone (**4**), and three known diterpenes: *ent*-16α-hydroxy-atisane-3-one (**1**), *ent*-atisane-3β,16α-diol (**2**) and *ent*-3,4-*seco*-16α-hydroxyatis-4(19)-en-3-oic acid (**3**) were isolated from the bark of *E. agallocha*. The structures of these known diterpenes were identified by comparison of their spectral data with those reported in the literature [19,20,21,22,23].

**Figure 2:**
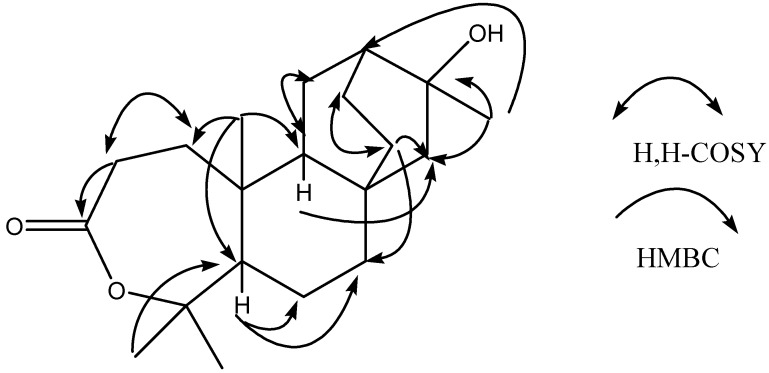
The key H, H-COSY and HMBC correlations of compound **4**.

Compound **4** was isolated as colorless needles, mp 153-154°C, [α]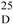: -40°(*c*=1.0, MeOH). The molecular formula of C_20_H_32_O_3_ was established by HR-ESI-MS at *m*/*z* 343.22502 ([M+Na] ^+^). The ESI-MS of **4** showed quasi-molecular ion peaks at *m*/*z* 343 ([M+Na] ^+^). Analysis of the ^1^H-NMR, ^13^C- NMR, DEPT and HMQC spectral data (Table 1) revealed the presence of four methyls (δc 30.0, 32.7, 24.6, 14.4), eight methylenes, three methines and five quaternary carbons (including a carbonyl group at δc 175.1 and two oxygenated carbons at δc 86.0, 71.3). The NMR spectra of **4** were similar to those of **2**, except for the fact that the peaks of C-3 (*δ*_C_ 175.1, s) and C-4 (*δ*_C_ 86.0, s) were shifted upfield, compared with those of **2 ** (*δ*_C-3_ 38.7, s and *δ*_C-4_ 79.1, s), indicating that the oxygenated methine (C-3) and the quaternary carbon (C-4) of **2** were replaced by a carbonyl group (C-3) and an oxygenated quaternary carbon (C-4). The peak of C-4 (*δ*_C_86.0, s) was shifted upfield compared with the carbon bearing OH group at *δ*_C_ 79.1 but was similar to the carbon bearing a lactone group at *δ*_C_ 84.8 [24]. This revealed the lactone between C-3 and C-4. Furthermore, H-2 showed long-correlations with a quaternary carbon at δ 175.1 (C-3), a methylene at δ 35.3 (C-1), and a quaternary carbon at δ 40.0 (C-10), H-12 showed long-range correlations with δ 49.7 (C-9), δ 26.3 (C-14), and δ 30.0 (C-17), H-14 showed long-range correlations with δ 49.7 (C-9), δ 56.6 (C-15), and δ 38.3 (C-7). In addition, other correlations for the quaternary carbons (C-4, C-8, C-10 and C-16) were also observed in the HMBC spectrum (Table 1). The H, H-COSY spectra revealed the connectivity between C-1/C-2, C-9/C-11 and C-13/C-14 (Figure 2, Table 1).

**Table 1:** ^13^C-NMR (150 MHz), ^1^H-NMR (600 MHz) data, HMBC and H, H-COSY correlations of Compound **4** (CDCl_3_).

No.	^13^C	^1^H	HMBC	H, H-COSY
1	35.3 t	1.72, m	175.1, 55.7, 49.7, 40.0, 31.2, 14.4	2.64
		1.47, m	175.1, 49.7, 40.0, 31.2, 14.4	2.64
2	31.2 t	2.64, m	175.1, 40.0, 35.5	1.72, 1.47
3	175.1 s			
4	86.0 s			
5	55.7 d	1.63, dd, (3.9, 10.4)	86.0, 49.7, 40.0, 38.3, 35.3, 32.7, 24.6, 22.1, 14.4	
6	22.1 t	1.49, m	55.7, 40.0, 38.3, 33.2	
7	38.3 t	1.39, m	56.6, 49.7, 33.2, 26.3, 22.1	
		1.20, m	56.6, 49.7, 33.2, 26.3, 22.1	
8	33.2 s			
9	49.7 d	1.35, ddd, (1.5, 7.0, 11.4)	56.6, 40.0, 35.3, 33.2, 26.3, 23.3, 14.4	2.09
10	40.0 s			
11	23.3 t	2.09, m	71.3, 49.7, 40.0, 37.5, 33.2, 23.2	1.35
		1.18, m	71.3, 37.5	
12	37.5 d	1.58, m	71.3, 56.6, 49.7, 30.0, 26.3, 23.3	
13	23.2 t	1.69, m	71.3, 37.5, 33.2, 26.3, 23.3	1.84, 0.87
		1.48, m	71.3, 37.5, 33.2, 26.3, 23.3	1.84, 0.87
14	26.3 t	1.84, m	56.6, 37.5, 33.2, 23.2	1.69, 1.48
		0.87, m	56.6, 49.7, 33.2, 23.2	1.69, 1.48
15	56.6 t	1.35, m	71.3, 49.7. 38.3, 33.2, 30.0, 26.3	
		1.24, m	71.3, 49.7. 38.3, 33.2, 30.0, 26.3	
16	71.3 s			
17	30.0 q	1.31, s	71.3, 56.6, 37.5	
18	32.7 q	1.46, s	86.0, 55.7, 24.6	
19	24.6 q	1.45, s	86.0, 55.7, 32.7	
20	14.4 q	1.17, s	55.7, 49.7, 40.0, 35.3	

**Figure 3:**
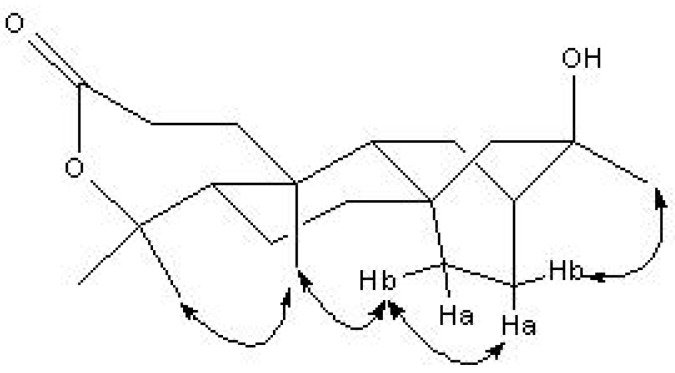
The key NOE correlations of compound **4**.

The above-mentioned evidence suggests that compound **4** has a tetracyclic atisane-type diterpenoid ring system with a lactone between C-3 and C-4. The relative stereochemistry of **4** was established by the NOESY correlations shown in Figure 3 and the comparison of its NMR data with that of compounds **1**, **2** and **3**. Comparison of the NMR data of **4** with **1**, **2** and **3** showed that the Me-17 (30.0, q), Me-19 (24.6, q) and Me-20 (14.4, q) were in a β-orientation. NOEs were detected between the signals of Me-17 and H-13b (δ_H_ 1.69, m), H-13a (δ_H_ 1.49, m) and H-14b (δ_H_ 1.84, m), H-14b (δ_H_ 1.84, m) and Me-20, Me-20 and Me-19, which indicated the same β-orientation of Me-17, Me-19 and Me-20. The absolute configuration of **4** was tentatively assumed to possess the same *ent* configuration of compounds **1**, **2** and **3** from the co-occurrence and close similarity of their structures and based on their negative sign of optical rotation. Consequently, the structure of **4** was determined to be *ent*-16α-hydroxy-atisane-3,4-lactone.

**Figure 4:**
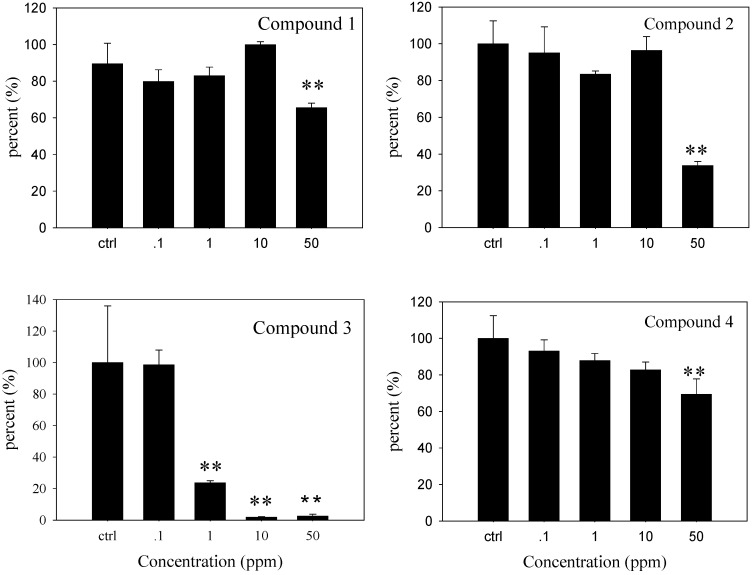
Adherence inhibition activity against the marine microorganism *P. pseudoalcaligenes* by compounds **1-4**. Data were analyzed using one-way ANOVA, where **P < 0.01 were significantly different from the control.

The anti-microfouling activities of the four compounds are shown in Figure 4. Pseudomonas were the best studied biofilm forming bacterial species[25]. *P. pseudoalcaligenes* was isolated from marine substratum and adhered firmly on a man-made surface. At 1 ppm (*P* < 0.01) compound **3** significantly reduced the adherence of *P. pseudoalcaligenes* compared to the negative control (a blank with sterile seawater, 0.5 mL DMSO and bacterial suspension without tested compound). Compounds **1**, **2** and **4** showed significant effects at 50 ppm (*P* < 0.01). Compound **3** showed anti-microfouling activity with EC_50_ values of 0.54 ± 0.01 ppm, which was lower than that of positive control CuSO_4_ (EC_50_ = 4.58 ± 0.37 ppm). Compound **2** showed activity with EC_50_ values of 29.5 ± 0.6 ppm. Compounds **1** and **4** inhibited adherence of *P. pseudoalcaligenes* with EC_50_ values above 50 ppm. 

Our structure-activity relationship analysis of atisane-type diterpenes suggested that the presence of a free hydroxyl group might be more important in the expression of the activity than a carbonyl group. Compound **2**, with a free hydroxyl group at C-3, had stronger activity than compound **1** with a carbonyl group at C-3, and compound **3**, a secoatisane-type diterpenoid with a carboxylic acid at C-3 had higher activity than compounds **1**, **2** or **4.**

## Experimental

### General

The ^1^H-, ^13^C- and 2D-NMR spectra were recorded with a Bruker Avance-600 FT NMR spectrometer (Germany). Low-resolution ESI mass spectra data were recorded on an AB 3200Q TRAP spectrometer (USA). HR-ESI mass spectra data were recorded on an APEXIII 7.0 TESLA FTMS from Bruker Daltonics, Inc (USA). The optical rotation data were obtained on a Rudolph Autopol IV Polarimeter (USA). Column chromatography was performed with silica gel (200-300 mesh), and GF254 silica gel for TLC was obtained from Qingdao Marine Chemistry Co. Ltd (Qingdao, P.R. China). ODS (Octadecyltrichlorosilane) and Sephadex LH-20 (18-110 μm) were obtained from Pharmacia Co (Sweden).

### Extraction and Isolation

The bark of *E. agallocha* was collected from Hainan Province, China in July 2006. The plant material was indentified by Professor Yi-Ming Lin, Xiamen University. A voucher specimen (HQ-2006-6) is deposited at the herbarium of the Department of Biology, School of Life Sciences, Xiamen University. The air-dried and powdered material (2.1 kg) was extracted with 95% EtOH three times at reflux temperature. Removal of the solvent from the combined EtOH extracts yielded a brown viscous mass (104 g). The extract was suspended in H_2_O, and partitioned with petroleum ether, EtOAc, and *n*-BuOH. The petroleum ether layer (32 g) was chromatographed on silica gel column with petroleum ether-EtOAc as gradient eluent to obtain eight fractions, 1-8. Fraction 3 (890 mg) was subjected to ODS column chromatography [MeOH-H_2_O (4:1) and MeOH] to yield fractions 3-1 and 3-2. Fraction 3-1 was subjected to silica gel column chromatography (petroleum ether-EtOAc = 18:1) to give compound **1** (27 mg). The EtOAc layer (15 g) was subjected to ODS column chromatography [MeOH-H_2_O (4:1) and MeOH] to give fractions 1 and 2. Fraction 1 was subjected to Sephadex LH-20 chromatography [MeOH-H_2_O (4:1) and MeOH] to give two fractions 1-1 and 1-2. Fraction 1-1 was purified by repeated silica gel column chromatography (petroleum ether-ethyl acetate) to yield compounds **2** (32 mg), **3** (7.2 mg) and **4**(18 mg).

*Compound* **1**: C_20_H_32_O_2_, colorless needles, mp 143-144 °C and [α]_D_ -29.0° (*c*=1.0, CHCl_3_) [lit. [20] mp 157-158 °C; [α]_D_ -33.0° (*c*=0.10, CHCl_3_)]; ESI-MS^+^ (m/z): 631 (2M+Na)^+^, 343 (M+K)^+^, 327 (M+Na)^+^; ^1^H-NMR (CDCl_3_, 600 MHz): *δ*1.38 (each 1H, m, H-1), 2.58, 2.33 (each 1H, m, H-2), 1.86, 12.9 (1H, m, H-5), 1.51, 1.39 (each 1H, m, H-6), 1.42, 1.16 (each 1H, m, H-7), 1.27 (1H, m, H-9), 2.05, 1.22 (each 1H, m, H-11), 1.56 (1H, m, H-12), 1.69, 1.52 (each 1H, m, H-13), 1.86, 0.86 (each 1H, m, H-14), 1.38, 1.21 (each 1H, m, H-15), 1.30 (3H, s, H-17), 1.08 (3H, s, H-18), 1.04 (3H, s, H-19), 1.11 (3H, s, H-20). ^13^C-NMR (CDCl_3_, 150 MHz): δ 37.9 (C-1), 34.0 (C-2), 217.5 (C-3), 47.6 (C-4), 55.6 (C-5), 19.5 (C-6), 38.7 (C-7), 33.5 (C-8), 50.2 (C-9), 37.1 (C-10), 23.1 (C-11), 37.8 (C-12), 23.8 (C-13), 26.9 (C-14), 57.1 (C-15), 71.9 (C-16), 30.5 (C-17), 21.6 (C-18), 26.1 (C-19), 13.4 (C-20).

*Compound* **2**: C_20_H_34_O_2_, colorless needles, mp 205-206 °C and [α]_D_ -28.0° (*c*=0.22, CHCl_3_); ESI-MS^+^ (m/z): 635 (2M+Na)^+^, 345 (M+K)^+^, 329 (M+Na)^+^; ^1^H-NMR (CDCl_3_, 600 MHz): *δ* 3.21 (1H, dd, *J*=10.8, 5.4 Hz, H-3), 1.28 (3H, s, H-17), 0.79 (3H, s, H-18), 0.98 (3H, s, H-19), 0.94 (3H, s, H-20). ^13^C-NMR (CDCl_3_, 150 MHz): δ 37.5 (C-1), 26.8 (C-2), 79.1 (C-3), 38.7 (C-4), 55.3 (C-5), 18.4 (C-6), 39.4 (C-7), 33.6 (C-8), 51.1 (C-9), 37.3 (C-10), 23.2 (C-11), 37.8 (C-12), 24.0 (C-13), 27.1 (C-14), 57.4 (C-15), 72.1 (C-16), 30.4 (C-17), 15.5 (C-18), 28.1 (C-19), 13.9 (C-20).

*Compound* **3**: C_20_H_32_O_3_, colorless plates, mp 101-102 °C and [α]_D _ -53.7°(*c*=1.0, CHCl_3_); ESI-MS^+^ (m/z): 663 (2M+Na)^+^, 359 (M+K)^+^, 343 (M+Na)^+^; ^1^H-NMR (CDCl_3_, 600 MHz): *δ* 4.81, 4.66 (each 1H, br s, H-19), 2.40 (2H, dd, *J*=9.0, 9.0 Hz, H-2), 1.73 (3H, s, H-18), 1.31 (3H, s, H-17), 0.95 (3H, s, H-20); ^13^C-NMR (CDCl_3_, 150 MHz): δ 32.8 (C-1), 27.5 (C-2), 177.6 (C-3), 147.5 (C-4), 50.5 (C-5), 24.5 (C-6), 38.0 (C-7), 33.5 (C-8), 42.0 (C-9), 39.3 (C-10), 23.2 (C-11), 37.7 (C-12), 23.8 (C-13), 26.7 (C-14), 56.2 (C-15), 73.4 (C-16), 30.2 (C-17), 23.6 (C-18), 113.2 (C-19), 17.8 (C-20).

*Compound **4***: C_20_H_32_O_3_*,* colorless needles, mp 153-154 °C and [α] _D_^25^ -40°(*c*=1.0, MeOH). ESI-MS^+^ (m/z): 359 (M+K)^+^, 343 (M+Na)^+^; HR-ESI-MS(m/z): 343.22502 (M+Na) ^+^; ^1^H-NMR and ^13^C-NMR data for **4** are listed in Table 1.

### Anti-microfouling Assay

The experimental method, adapted to screen antifouling agents, was based on bacterial adhesion in natural sterile sea water in a microtiter plate and on total biomass quantification by the fluorescent dye 4,6-diamidino-2-phenylindole (DAPI) [26]. The adhered microorganisms, *P. pseudoalcaligenes*, were incubated in a 50-mL flask containing 2216E liquid medium at 30 °C for 12 h. The mature biofilms were incubated with different concentrations of compounds in 6-well plates with a cover glass at 30 °C for 3 h. The cover glasses were washed with water. And these glasses were observed by microscope after dying with 4 µg/mL DAPI for 20 min, and assayed. Differences in the settlement percentages of experimental and control treatments were tested for significance by one-way ANOVA. Significance was set at the 1% level. The EC50 (the concentration that reduces the settlement rate by 50% relative to the control) was estimated by using the Spearman-Karber method [27].
